# Familial Hypercholesterolemia: A Systematic Review of Guidelines on Genetic Testing and Patient Management

**DOI:** 10.3389/fpubh.2017.00252

**Published:** 2017-09-25

**Authors:** Giuseppe Migliara, Valentina Baccolini, Annalisa Rosso, Elvira D’Andrea, Azzurra Massimi, Paolo Villari, Corrado De Vito

**Affiliations:** ^1^Department of Public Health and Infectious Diseases, Sapienza University of Rome, Rome, Italy

**Keywords:** familial hypercholesterolemia, systematic review, guidelines, genetic testing, cascade screening

## Abstract

**Background:**

Familial hypercholesterolemia (FH) is an autosomal-dominant hereditary disorder of lipid metabolism that causes lifelong exposure to increased LDL levels resulting in premature coronary heart disease and, if untreated, death. Recent studies have shown its prevalence to be higher than previously considered, which has important implications for the mortality and morbidity of associated cardiovascular disease (CVD). Several clinical tools are used worldwide to help physicians diagnose FH, but nevertheless most patients remain undetected. This systematic review of guidelines aims to assess the role of genetic testing in the screening, diagnosis, and management of patients affected by heterozygous or homozygous FH and to identify related health-care pathways.

**Methods:**

We performed a systematic review of the literature; inclusion criteria were English or Italian guidelines focusing on genetic testing. The guidelines were included and evaluated for their content and development process using the Appraisal of Guidelines for Research and Evaluation II instrument.

**Results:**

Ten guidelines were considered eligible, and all were judged to be of good quality, with slight differences among them. The most common indications for performing genetic tests were high levels of cholesterol, or physical findings consistent with lipid disorder, in the subject or in the family history. Subsequent screening of family members was indicated when a mutation had been identified in the index patient. Regarding patient management, the various guidelines agreed that intensive treatment with lipid-lowering medications should begin as quickly as possible and that lifestyle modifications should be an integral part of the therapy.

**Conclusion:**

Since the early detection of affected patients is beneficial for effective prevention of CVD, genetic testing is particularly useful for identifying family members *via* cascade screening and for distinguishing between heterozygous and homozygous individuals, the latter of which require more extreme therapeutic intervention.

## Introduction

Familial hypercholesterolemia (FH) is an autosomal-dominant genetic disorder of lipid metabolism which leads to markedly elevated plasma concentrations of low-density lipoprotein cholesterol (LDL-C) (1). Long exposure to high levels of circulating LDL accelerates atherosclerotic cardiovascular disease (CVD) and especially coronary heart disease (CHD). If left untreated, men and women with heterozygous FH typically develop CHD before age of 55 and 60 years, respectively (2), while individuals with homozygous FH typically develop CHD before they are 20 years old and do not survive beyond age 30 (3). In 79% of cases, FH is caused by mutations in the LDL receptor (LDLR) gene, resulting in defective synthesis, assembly, transport, and recycling of the LDLR or in impaired endocytosis of LDLs. Apolipoprotein B (APOB) helps the LDLR bind LDL, while proprotein convertase subtilisin/kexin type 9 (PCSK9) degrades the LDLR; mutations in the respective APOB and PCSK9 genes account for 5 and <1% of FH cases, respectively. The remaining 15% of FH cases are either polygenic or are caused by other rare monogenic mutations in the APOE, SREBP2, and STAP1 genes (4). A very rare recessive form of FH is caused by mutation in the LDLRAP1 gene (5).

The majority of affected individuals are heterozygous [heterozygous familial hypercholesterolemia (HeFH)] and, according to recent studies (6–8), the prevalence of HeFH is higher than previously thought: it is now believed that it affects between 1/200 and 1/300 subjects and thus it is the most common monogenic disorder. In contrast, the global prevalence of homozygous individuals [homozygous familial hypercholesterolemia (HoFH)] is much lower, occurring in 1 in every 160,000–300,000 subjects (4). Homozygous patients can have the same mutation in both alleles of the same gene, or more commonly, they are compound heterozygotes with different mutations in each allele of the same gene, or, finally, they can be double heterozygotes with mutations in two different genes affecting LDLR function.

Identification of FH patients can be achieved by clinical diagnosis, by examination of personal and family history, or by genetic testing. The key characteristics of the disorder, which are elevated plasma LDL-C concentration, tendon xanthomas or corneal arcus, and family history of premature CHD, have been used to develop the most widely applied tools to support physicians during diagnosis. These are the Dutch Lipid Clinic Network (DLCN) criteria, the Make Early Diagnosis to Prevent Early Death (MEDPED) criteria, and the Simon Broome Register (SBR) criteria (9). The MEDPED criteria rely on age-specific and family relative-specific total cholesterol levels only, while the DLCN and the SBR both include a number of other, similar criteria; nevertheless, no standardized international tools currently exist.

Genetic testing can confirm a clinical diagnosis or assist in identifying individuals whose close relatives will subsequently require screening; since FH is a disease inherited in an autosomal-dominant manner, cascade screening is a highly cost-effective means of identifying at-risk individuals by a process of systematic family tracing (10). Several types of genetic test are available, which adopt different approaches. The most rapid tests aim to identify a specific mutation in the LDLR, APOB, or PCSK9 genes that has already been identified in another family member (11). At the opposite, extreme are tests that check for all known and possible mutations in recognized disease genes [i.e., next-generation sequencing (NGS) for comprehensive mutation detection or in specific loci of interest] (12).

Although current DNA testing has demonstrated high levels of specificity and sensitivity, especially when combined with clinical criteria, the failure to find a mutation does not necessarily exclude a diagnosis of FH (13). There is therefore a need to improve the early detection of FH, which is essential for effective reduction of the morbidity and mortality of CVD patients. This is particularly pertinent given the scale of the problem: it has been estimated that there are between 14 and 34 million individuals with FH worldwide (14, 15), but that less than 1% of potential patients have been identified in many countries (15).

Therefore, this systematic review of guidelines aims to evaluate the role and importance of genetic testing in the screening, diagnosis, and management of FH patients and summarizes related health-care pathways.

## Methods

To find existing guidelines on FH diagnosis and management, a systematic review of the literature was performed using the PubMed database and Google Scholar between March and April 2017 with the search string (“Guidelines as Topic” [Mesh] OR “Practice Guidelines as Topic” [Mesh] OR “Guideline” [Publication Type] OR “guideline” OR “guidelines”) AND (“Hyperlipoproteinemia Type II” [Mesh] OR “Hyperlipoproteinemia Type III” [Mesh] OR “Familial Hypercholesterolemia”) for PubMed, and Guidelines and “Familial Hypercholesterolemia” for Google Scholar. In addition, the websites of the leading national and international scientific societies operating in the field of cardiovascular risk (CVR) control were searched for guidelines on FH (see Table S1 in Supplementary Material for a list of the websites). The articles were retrieved from electronic databases and websites, and duplicates were removed. After screening titles, some further articles were excluded. The remaining articles were considered eligible if the guidelines focused on genetic testing and its role in the diagnosis, screening, and management of FH patients and were in English or Italian. Where multiple guidelines were obtained from the same scientific society, the most recent was retained. Two authors independently extracted results from the retrieved guidelines, and disagreements were resolved by a third author (16). Appraisal of Guidelines for Research and Evaluation II (AGREE II)—currently the most widely endorsed appraisal tool (16, 17)—was used to assess the quality of the guidelines by two authors independently. AGREE II consists or 23 key items organized within 6 domains (Scope and Purpose, Stakeholder involvement, Rigor of Development, Clarity of Presentation, Applicability, and Editorial Independence). Each item is rated on a 7-point scale (being 1 as strongly disagree and 7 as strongly agree) (16). PRISMA guidelines for reporting of systematic reviews and meta-analysis were followed (18).

## Results

### Characteristics of the Guidelines

**Figure 1:**
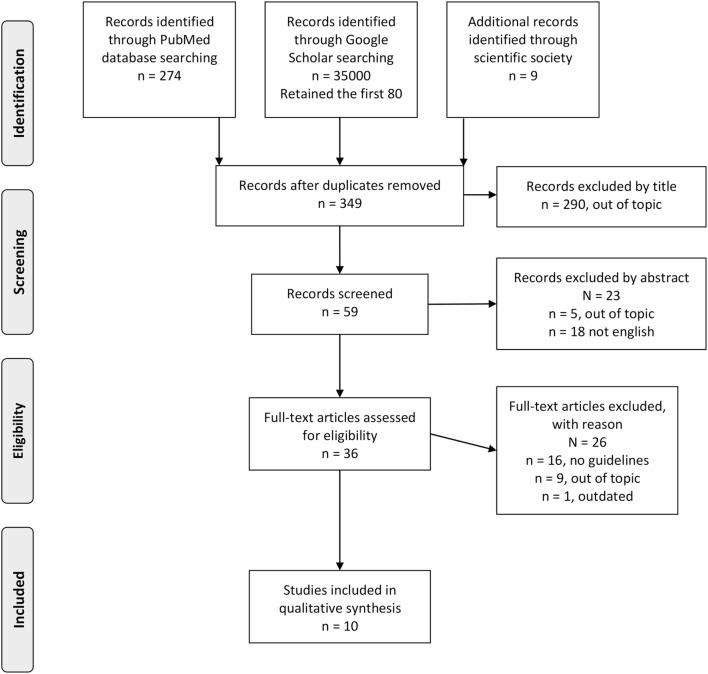
Flow diagram of the study selection process.

Overall, 10 guidelines met the inclusion criteria and were included in our systematic review (see Figure 1 and Table 1). Three guidelines were drafted by international panels (19–21), three by panels in the USA (9, 22, 23), and one guideline each by Italian (24), Belgian (25), British (26), and Taiwanese (27) panels. These were published between 2011 (21, 23, 25) and 2017 (22, 27). Four guidelines (20–22, 27) declared editorial independency, three contained claims about conflicts of interest (9, 19, 23), and three did not make a declaration (24–26). According to AGREE II, the average overall score was 4.6, ranging from 6 (9) to 3 (24). The domain with the worst average percentage of agreement was Applicability (34.3%), followed by Rigor of Development (36.8%), Editorial Independence (38%), and Stakeholder Involvement (46.9%). The domains with higher compliance were Clarity of Presentation (80.2%) and Scope and Purpose (82.7%).

**Table 1:** Characteristics of the guidelines.

Guidelines, year	Organization responsible for guidelines development	Country applied	Conflict of interests	AGREE II overall assessment
Familial Hypercholesterolemia; screening, diagnosis and management of pediatric and adult patients, 2011 (23)	National Lipid Association (NLA)	USA	SCI	4

Familial Hypercholesterolemia: A Model of Care for Australasia, 2011 (21)	Familial Hypercholesterolemia Australasia Network Consensus Group (FHANCG)	Oceania	EI	5

Management of Familial Hypercholesterolemia in Children and Young Adults: Consensus Paper Developed by a Panel of Lipidologists, Cardiologists, Paediatricians, Nutritionists, Gastroenterologists, General Practitioners and a Patient Organization, 2011 (25)	NA (Descamps)	Belgium	NA	5

Integrated guidance on the care of familial hypercholesterolemia from the International FH Foundation, 2014 (9)	International FH Foundation (IFHF)	USA	SCI	6

Linee guida cliniche per la prevenzione della cardiopatia ischemica nella ipercolesterolemia familiare: una patologia sotto-diagnosticata e sotto-trattata, 2014 (24)	Società Italiana Studio Aterosclerosi (SISA)	Italy	NA	3

Identification and Treatment of Patients with Homozygous Familial Hypercholesterolaemia: Information and Recommendations from a Middle East Advisory Panel, 2015 (19)	NA (Al-Ashwal)	Middle East	SCI	5

Familial hypercholesterolemia: identification and management, 2016 (26)	National Institute for Health and Care Excellence (NICE)	UK	NA	4

Guidelines for the Management of Dyslipidemia, 2016 (20)	European Society of Cardiology and the European Atherosclerosis Society (ESC-EAS)	Europe	EI	5

Taiwan lipid guidelines for high risk patients, 2017 (27)	Taiwan Society of Lipids and Atherosclerosis (TSLA)	Taiwan	EI	4

Guidelines for Management of Dyslipidemia and Prevention of Cardiovascular Disease, 2017 (22)	American Association of Clinical Endocrinologists and American College of Endocrinology (AACE-ACE)	USA	EI	5

*EI, editorial independence declared; FIP, funding by industrial partners reported; FPO, funding by external public organization reported; SCI, statement about conflict of interests of group members present; AGREE II, Appraisal of Guidelines for Research and Evaluation II*.

### Genetic Testing

Table 2 describes the characteristics that identify subjects requiring genetic testing for FH, according to the various guidelines. Six guidelines (9, 19, 21, 23, 25, 26) indicate to search for mutations of LDL-R, APOB, and PCSK9 genes, while the others do not specify which detection method is recommended. One guideline (23) asserts that there is no need to screen the general population. In children, genetic analysis is indicated when cholesterol levels are high, according to three guidelines (19, 20, 24), although these guidelines differ in cutoff values and whether to take into account both total cholesterol and LDL-C. Six guidelines (9, 20, 21, 24–26) indicate DNA testing of a child whose parent has a confirmed diagnosis of FH. One guideline (9) suggests performing DNA analysis even in the case of dead or unknown parents. For adults, five guidelines recommend genetic testing where individuals present with high total cholesterol or LDL-C levels (see Table 2) (20, 22–24, 27). Three guidelines (9, 20, 24) indicate a genetic test to confirm clinical diagnosis of FH and four guidelines (9, 20, 24, 27) where there are clinical manifestations of FH (i.e., xanthoma and/or premature CHD/CVD). Six guidelines (9, 20, 23, 24, 26, 27) recommend DNA testing in the case of family history of FH, while two guidelines (22, 23) indicate this even if the family history is only suggestive of FH or where there is a family history of xanthoma or premature CHD/CVD. Six guidelines (9, 20, 23, 24, 26, 27) indicate the use of DNA testing for cascade screening. Only one guideline (19) suggests that genetic counseling should be offered to couples at risk of HoFH in their offspring. In consideration that only about 80% of the tested patients results positive for a mutation in LDL-R, APOB, or PCSK9 gene, a negative test does not exclude the diagnosis of FH. Seven guidelines (9, 19, 21, 23, 25, 26) specifically recommend to consider the patients negative to genetic test which fall within clinical criteria for a possible FH diagnosis.

**Table 2:** Criteria to select individuals for genetic testing.

Target population	Characteristics	Recommendation	Guidelines, year	Strength of recommendation
General population		DNA testing is not needed	NLA, 2011 (23)	NA

	During acute illness or use of statins	DNA testing is not indicated	IFHF, 2014 (9)	2A

	With an unlikely phenotypic diagnosis of FH	DNA testing is not needed	FHANCG, 2011 (21) IFHF, 2014 (9)	C 1C

Children	With cholesterol concentration >230 mg/dl or >95° percentile for age and sex	DNA testing is indicated	SISA, 2014 (24)	NA

	With LDL concentration >150 mg/dl	DNA testing is indicated	ESC-EAS, 2016 (20)	1C

	With low-density lipoprotein cholesterol (LDL-C) concentration >500 mg/dl in untreated patients	DNA testing is indicated	Al-Ashwal, 2015 (17)	NA

	With LDL-C concentration >300 mg/dl in treated patients	DNA testing is indicated	Al-Ashwal, 2015 (17)	NA

	With a parent with FH	DNA testing is indicated	FHANCG, 2011 (21) Descamps, 2011 (25) SISA, 2014 (24) IFHF, 2014 (9) ESC-EAS, 2016 (20) NICE, 2016 (26)	A 1C NA 1A 1C NA

	With parents deceased or unknown	DNA testing is indicated	IFHF, 2014 (9)	3B

	With xanthoma or other physical findings of homozygous FH or at risk of homozygous FH	DNA testing is indicated by 2 years of age	IFHF, 2014 (9)	2A

	With suspected heterozygous FH	DNA testing is indicated between the ages of 5 and 10	IFHF, 2014 (9) ESC-EAS, 2016 (20)	2B 1C

	With suspected homozygous FH	DNA testing is indicated earlier than 5 years of age	ESC-EAS, 2016 (20)	1C

	Based on age- and gender-specific LDL-C levels	DNA testing is indicated ideally before puberty	IFHF, 2014 (9) IFHF, 2014 (9)	2B 2C

Adult patients	With cholesterol concentration >310 mg/dl or >95° percentile for age and sex	DNA testing is indicated	SISA, 2014 (24)	NA

	With LDL concentration >190 mg/dl	DNA testing is indicated	ESC-EAS, 2016 (20) NLA, 2011 (23)	NA NA

	With a known family history of FH	DNA testing is indicated	NLA, 2011 (23) SISA, 2014 (24) IFHF, 2014 (9) ESC-EAS, 2016 (20) NICE, 2016 (26) TSLA, 2017 (25)	NA NA 1A 1C NA 1C
		Pretesting counseling should be offered prior to any form of testing	IFHF, 2014 (9)	1A

	With a known family mutation	DNA testing for cascade screening is indicated	NLA, 2011 (23) IFHF, 2014 (9) SISA, 2014 (24) ESC-EAS, 2016 (20) NICE, 2016 (26) TSLA, 2017 (25)	NA 1A NA 1C NA 1C

	With a family history equivocal or only suggestive of FH	DNA testing is indicated	NLA, 2011 (23)	NA

	With a family history of high cholesterol levels (total, non-HDL and LDL) consistent with FH	DNA testing is indicated	AACE-ACE, 2017 (22)	4C

	With a clinical diagnosis of FH	DNA testing is indicated	NLA, 2011 (23) FHANCG, 2011 (21) IFHF, 2014 (9)	NA A 3A

	With >5 points on the Dutch Score	DNA testing is indicated	SISA, 2014 (24)	NA

	With diagnosis of xanthoma or coronary heart disease (CHD) in the family history	DNA testing is indicated	SISA, 2014 (24) ESC-EAS, 2016 (20)	NA 1C

	With a family history of unlikely diagnosis of FH	DNA testing is not needed	IFHF, 2014 (9)	1C

	With severe hypercholesterolemia, tendon xanthoma, and/or premature CAD	DNA testing is indicated	TSLA, 2017 (25)	1C

	With CHD or cardiovascular disease before the age of 55 years for men and 60 years for women	DNA testing is indicated	SISA, 2014 (24) IFHF, 2014 (9) ESC-EAS, 2016 (20)	NA 1A 1C

	With premature ASCVD (MI or sudden death) before age of 55 years in father or other male first-degree relative, or before age 65 years in mother or other female first-degree relative	DNA testing is indicated	AACE-ACE, 2017 (22) SISA, 2014 (24)	4C NA

	Couples where there is a risk of homozygous familial hypercholesterolemia in their offspring	Genetic counseling	Al-Ashwal, 2015 (19)	NA

### Management of Positive Patients

Table S2 in Supplementary Material describes the health-care pathways that should be provided to patients positive for HeFH and HoFH, grouped for adult patients, woman, and children. Due to the large number of recommendations, we report below only the most relevant ones (see Table S2 in Supplementary Material for a comprehensive list of recommendations in the guidelines).

#### Adults

Regarding non-pharmacological treatment, six guidelines (9, 21–24, 26) recommend counseling adult patients about lifestyle modification; three of them (23, 24, 26) suggest taking at least 30 min of physical activity a day, while four (22–24, 26) advise patients to stop smoking and to achieve and maintain a healthy weight. Three guidelines (22, 23, 26) contain recommendations on nutrition habits. Six guidelines (9, 20, 22–24, 26) point out that all adult patients with HeFH should receive lipid-modifying drug treatment to reduce LDL-C; three guidelines (22, 23, 26) recommend statin therapy as the primary pharmacological means of achieving target LDL levels; and six guidelines (9, 21–24, 26) recommend a combination therapy when LDL-C targets are not achieved. Four guidelines (9, 21, 22, 26) recommend monitoring indicators of liver and kidney injury during pharmacological treatment. Three guidelines (9, 23, 26) suggest LDL apheresis in patients under maximal drug therapy who still cannot achieve the LDL-C target; two guidelines (21, 23) recommend LDL apheresis when CVR is very high and LDL-C is above 160 mg/dl; and one guideline (23) suggests LDL apheresis in patients with high CVR and LDL-C above 200 mg/dl, or with LDL-C above 300 mg/dl even without other CVRs. Regarding LDL-C targets, five guidelines (9, 20, 23–25) recommend a target of LDL-C < 100 mg/dl for patients without other CVR [or even with high CVR in the case of two guidelines (23, 24)], while one guideline (22) recommends a target of <70 mg/ml; five guidelines recommend a target LDL-C level of <70 mg/dl for patients with very high CVR (9, 20, 24, 27) or with clinical CVD (9, 24). The AACE/ACE guideline recommends keeping LDL-C levels below 70 mg/ml in patients with clinical CVD (22). For HoFH patients, five guidelines (9, 27) recommend LDL apheresis, while four guidelines (9, 19, 24, 27) recommend a target LDL-C level of <70 mg/dl where clinical CVD is present in these patients.

#### Women

Four guidelines (9, 21, 23, 26) recommend that lipid-modifying therapy should not continue during attempts to conceive (see Table S2 in Supplementary Material for timetable) and three of these (9, 21, 23) suggest offering prepregnancy counseling. Only two guidelines (9, 26) suggest offering female patients advice on contraception. Three guidelines (19, 23, 26) recommend the immediate interruption of statin treatment in the case of pregnancy, and two of them (19, 26) suggest offering an assessment of CHD risk. During breast feeding, five guidelines (9, 19, 21, 23, 26) recommend that statin therapy be suspended, and one guideline suggests the use of resins (26). Two guidelines (19, 23) suggest starting LDL apheresis, but only for HoFH women.

#### Children

For children, the recommendations for LDL-C target include <160 mg/dl (21), <135 mg/dl (20, 27), and <100 mg/dl (22). Five guidelines recommend beginning statin therapy between 8 years (9, 20) and 10 years (21, 22, 26) of age, while four guidelines (9, 21, 22, 24) indicate to operate on lifestyle modifications and non-lipid risk factors. Three guidelines (9, 21, 26) also suggest routinely monitoring growth and pubertal development. Four guidelines (21, 23–25) recommend commencing statin therapy from 10 years of age only when other CVRs are present, and three of these (21, 23, 24) indicate a target serum concentration of LDL-C lower than 130 mg/dl, while one (25) suggests a reduction of at least 30%. Seven guidelines (9, 19, 21, 23, 24, 26, 27) contain specific recommendations for HoFH children: three (19, 23, 27) indicate a target LDL-C concentration in serum lower than 135 mg/dl and the use of statins as primary intervention with or without ezetimibe. Moreover, four guidelines (9, 21, 23, 24) recommend LDL apheresis, which in two cases (9, 19) should be started by the age of 5 years and not after the age of 8 years.

## Discussion

In this systematic review of FH diagnosis and management guidelines, we focused on health-care pathways relating to genetic testing, both in referring individuals for testing and in recommending the correct preventive program triggered by test results and by familial or personal history. All the documents included in our search were evaluated using the AGREE II instrument and were assessed as average-to-good practice guidelines. Although they were judged to be adequate in their clarity of the purpose and in the exposition of the recommendations, major concerns surround the poor description of the methodology used to produce the recommendations in most of the guidelines and the lack of information about the funding received and the conflicts of interest. Even when a conflict of interest is declared, there is no description on how it is handled. Where DNA testing for FH is recommended, our results show that testing is indicated for early detection of FH and is especially useful for identifying family members of carrier patients. Therefore, genetic testing can help physicians find possible FH cases using an integrated model of diagnosis.

Although the various guidelines propose different LDL-C cutoff levels, the majority agree on genetic testing of potential FH carriers after cholesterol level has been measured or other physical findings distinctive of FH have been observed or, in some cases, where family history is highly suggestive of FH. Moreover, genetic analysis is recommended if the DLCN score is >5 but additional criteria are needed to confirm FH diagnosis. Only the SBR criteria accept the presence of a DNA mutation as a definitive confirmation of FH, whereas the MEDPED criteria do not even take genetic testing into account in the FH diagnosis (28). Nevertheless, performing universal screening of FH by genetic testing is not recommended in any of the guidelines, and one of them specifically advises against this possibility (23).

Once a causative mutation has been found in the index patient, DNA testing is highly indicated for first-degree family members, and possibly also for second- and third-degree relatives (9, 20, 23, 24, 26, 27), an approach that is reported to be highly cost-effective in finding FH patients (29, 30). Unfortunately, several authors in recent years have documented ineffective index case identification (15, 31, 32). In fact, available tools commonly used to aid FH diagnosis are believed to be insufficiently effective and, accordingly, a prognostic model to enhance FH detection in primary care has recently been developed (33). Furthermore, not finding a mutation does not rule out a diagnosis of FH, since the molecular techniques used are not 100% sensitive (13); this is in line with our results, which indicate that genetic analysis needs to be performed during the index patient assessment, when clinical criteria could underline a suspected FH case. It has been showed that the implementation of new techniques, such as the so-called NGS methodologies, may improve the detection rate of mutation causative of FH (34) with lower cost and labor associated compared to DNA testing with conventional sequencing (35). Studies using these techniques will have to be taken into account in future updating of guidelines since the guidelines included in this review did not stated it explicitly.

With regard to health-care pathways followed after a genetic test result, this systematic review highlights the importance of genetic testing in distinguishing between heterozygous and homozygous patients. The various guidelines adopt different LDL-C levels as therapy targets, but they are similar within each patient group, with the homozygous group requiring stricter therapies and lower LDL-C target levels than the heterozygous group. However, the majority of guidelines agree on the definition of progressive interventions based on the patients’ genetic status and LDL-C levels: LDL apheresis is the treatment of choice to immediately reduce cholesterol level, and it is essential for the HoFH patients’ management where conventional lipid-lowering drug therapies are usually not sufficient.

It is particularly relevant that DNA analysis is performed in children where knowing their genetic status could determine the endorsement of aggressive therapeutic strategies at an earlier age to prevent premature CHD; especially, HoFH children are at very high risk of developing CVD and therefore they need to be managed efficiently since the beginning.

In conclusion, this study highlights the importance of DNA testing for the identification of FH patients and their carrier status at the earliest opportunity, which has significant benefits and implications with respect to mortality and morbidity. Currently, the best approach to ensure an effective patients’ management may be represented by a combined strategy of genetic testing and clinical approach to achieve the highest level of accuracy in the FH case identification. In addition, once a mutation causative of FH has been found in the index patient, the cascade genetic screening using DNA analysis is an excellent tool to obtain an efficient detection of affected relatives. Indeed, while FH is a significant risk factor for CVD, it is also a treatable disorder whose inherited nature makes finding FH cases among family members of an index case essential. With this aim, further studies on how to improve detection of index cases could begin to address the need for early and effective management of FH patients and consequent arrest of the onset of premature CHD.

## Author Contributions

All the authors contributed to the concept and design of the study, are currently actively involved, and read and approved the final manuscript.

## Conflict of Interest Statement

The authors declare that the research was conducted in the absence of any commercial or financial relationships that could be construed as a potential conflict of interest.

## Abbreviations

FH, familial hypercholesterolemia; LDL-C, low-density lipoprotein cholesterol; CVD, cardiovascular disease; CHD, coronary heart disease; LDLR, low-density lipoprotein receptor; HeFH, heterozygous familial hypercholesterolemia; HoFH, homozygous familial hypercholesterolemia; DLCN, Dutch Lipid Clinic Network; MEDPED, Make Early Diagnosis to Prevent Early Death criteria; SBR, Simon Broome Register; AGREE II, Appraisal of Guidelines for Research and Evaluation II.
