# Supplementary material for: Familial Hypercholesterolemia: A Systematic Review of Guidelines on Genetic Testing and Patient Management
Source: Front Public Health. 2017 Sep 25;5:252. doi: 10.3389/fpubh.2017.00252 (PMC5622145; doi:10.3389/fpubh.2017.00252)
Supplement: Supplementary file 1 [file Table_1.DOCX]

**Supplentary Table 1. Website list of the leading national and international scientific societies searched.**

| **Society Name** | **Website** |
| --- | --- |
| American Association of Clinical Endocrinologists | http://www.aace.com/ |
| American Heart Association | http://www.heart.org/ |
| European Atherosclerosis Society | http://www.eas-society.org/ |
| European Society of Cardiology | http://www.escardio.org/ |
| International Atherosclerosis Society | http://www.athero.org/ |
| International FH Foundation | http://www.fh-foundation.org/ |
| National Institute for health and Care Excellence | http://www.nice.org.uk/ |
| National Lipid Association | http://www.lipid.org/ |
| Scottish Intercollegiate Guidelines Network | http://www.sign.ac.uk/ |
| Società Italiana Studio Aterosclerosi | http://www.sisa.it/ |
| Taiwan Society of Lipids and Atherosclerosis | http://www.tas.org.tw/ |
| The Cardiac Society of Australia and New Zealand | http://www.csanz.edu.au/ |
