# Supplementary material for: Familial Hypercholesterolemia: A Systematic Review of Guidelines on Genetic Testing and Patient Management
Source: Front Public Health. 2017 Sep 25;5:252. doi: 10.3389/fpubh.2017.00252 (PMC5622145; doi:10.3389/fpubh.2017.00252)
Supplement: Supplementary file 2 [file Table_2.DOCX]

Supplementary Table 2. Recommendations and strength of recommendation by results of genetic test.

| **CHARACTERISTICS** | **RECOMMENDATION** | **GUIDELINE, Y** | **STRENGTH OF RECOMMENDATION** |
| --- | --- | --- | --- |
| **adult patients with negative test result** | | |  |
| Phenotypic diagnosis of FH | The diagnosis of FH can’t be excluded | FHANCG, 2011 (25)  NLA, 2011 (23)  IFHF, 2014 (9)  SISA, 2014 (24) | A  NA  1A  NA |
|  | Comprehensive exon by exon sequencing is recommended | FHANCG, 2011 (25)  IFHF, 2014 (9) | A  1A |
| Family history with a known mutation of FH | Manage the coronary heart disease risk as in the general population | NICE, 2016 (26) | NA |
| **adult patients with positive (heterozygous) test result** | | |  |
|  | Should inform patients that the treatment is lifelong | NICE, 2016 (26) | NA |
|  | Should receive clear and appropriate educational information about FH and written advice or information about patient support groups | NICE, 2016 (26) | NA |
|  | Should encourage patients to contact their relatives to inform them of their potential risk | NICE, 2016 (26) | NA |
|  | Should be offered a regular structured review that is carried out at least annually; it includes update of the family pedigree, assessment of any symptoms of CHD, smoking status, a fasting lipid profile, discussion about concordance with medication, possible side effects | NICE, 2016 (26) | NA |
|  | A baseline ECG should be considered | NICE, 2016 (26) | NA |
|  | The 10-year CHD risk is not recommended | NLA, 2011 (23) | NA |
|  | Receive lipid-modifying drug treatment to reduce LDL concentration by more than 50% from baseline | NLA, 2011 (23)  SISA, 2014 (24)  IFHF, 2014 (9)  NICE, 2016 (26) | NA  NA  2C  NA |
|  | When the drug therapy does not achieve target LDL levels, referral to a specialist should be offered for consideration for treatment with either a bile acid sequestrant, nicotinic acid, or a fibrate | NICE, 2016 (26) | NA |
|  | fat-soluble vitamin (A, D, K) and folic acid supplementation should be offered to patients who are receiving long-term treatment with the bile acid sequestrants | NICE, 2016 (26) | NA |
|  | Receive lipid-modifying drug treatment to reach maximal LDL reduction | ESC-EAS, 2016 (20)  AACE-ACE, 2017 (22) | 2C  1A |
|  | Statin therapy is recommended as the primary pharmacologic agent to achieve target LDLc levels | NLA, 2011 (23)  NICE, 2016 (26)  AACE-ACE, 2017 (22) | NA  NA  1A |
|  | Statin with a low acquisition cost in people > 60 years of age and who do not have CHD | NICE, 2016 (26) | NA |
|  | Combination therapy of lipid-lowering agents should be considered when LDL target levels are not achieved | NLA, 2011 (23)  IHFH, 2014  AACE-ACE, 2017 (22) | NA  NA  1A |
|  | PCSK9 inhibitors should be considered for use in combination with statin therapy for LDLc lowering | AACE-ACE, 2017 (22) | 1A |
|  | Re-assess individuals’ lipid status 6 weeks after therapy initiation and again at 6-week intervals until treatment goal is achieved.  While on stable lipid therapy, individuals should be tested at 6-12 month intervals | AACE-ACE, 2017 (22) | 4D |
|  | Should be counselled regarding lifestyle modification | NLA, 2011 (23)  FHANCG, 2011 (25)  SISA, 2014 (24)  IFHF, 2014 (9)  AACE-ACE, 2017 (22)  NICE, 2016 (26) | NA  A  NA  2A  1A  NA |
|  | Maximize reduction in other ASCVD risk factors | NLA, 2011 (23) | NA |
|  | Take at least 30 minutes of physical activity a day | NLA, 2011 (23)  AACE-ACE, 2017 (22)  NICE, 2016 (26) | NA  1A  NA |
|  | Who is unable to perform moderate-intensity physical activity at least 5 days a week, should exercise at maximum safe capacity | NICE, 2016 (26) | NA |
|  | In addition to aerobic activity (4-6 times per week) muscle-strengthening activity is recommended at least 2 days a week | AACE-ACE, 2017 (22) | 1A |
|  | Achieve and maintain a healthy weight | NLA, 2011 (23)  SISA, 2014 (24)  NICE, 2016 (26) | NA  NA  NA |
|  | A reduced-calorie diet consisting of fruit and vegetables (5 portions per day), grains, fish and meat is recommended. | NICE, 2016 (26)  AACE-ACE, 2017 (22) | NA  1A |
|  | The intake of saturated fats, transfats, and cholesterol should be limited while LDLc lowering macronutrient intake should include plant stanols/sterols and fiber | AACE-ACE, 2017 (22) | 1A |
|  | Should be offered individualised nutritional advice from an expert on nutrition | NICE, 2016 (26) | NA |
|  | Should be advised to consume a diet in which:  total fat intake 30% or less of the total  saturated fats 10% or less of the total  intake of dietary cholesterol is less than 300 mg/day  saturated fats are replaced by increasing the intake of monounsaturated and polyunsaturated fats | NICE, 2016 (26) | NA |
|  | should not routinely recommended to take omega-3 fatty acid supplements | NICE, 2016 (26) | NA |
|  | Alcohol consumption should be limited to up 3-4 units a day for men and 2-3 for women | NLA, 2011 (23)  NICE, 2016 (26) | NA  NA |
|  | People who smoke should be advised to stop | NLA, 2011 (23)  SISA, 2014 (24)  NICE, 2016 (26)  AACE-ACE, 2017 (22) | NA  NA  NA  2A |
|  | People who want to stop smoking should be offered support and advice, also with pharmacotherapy | NICE, 2016 (26) | NA |
|  | Blood pressure should be treated to <140/90 or <130/80 in those with diabetes | NLA, 2011 (23) | NA |
|  | Plasma levels of hepatic aminotransferases, creatine kinase and creatinine should be measured before starting pharmacotherapy.  Hepatic aminotransferases should be monitored, creatine kinase should be measured when musculoskeletal symptoms are reported, creatinine should be monitored in those with kidney disease | FHANCG, 2011 (25)  IFHF, 2014 (9)  NICE, 2016 (26)  AACE-ACE, 2017 (22) | A  2A  NA  4C |
|  | Glucose should be monitored when there are risk factors for diabetes | IFHF, 2014 (9) | 2A |
|  | Mild elevations in blood glucose levels and or increased risk of T2DM associated with intensive statin therapy do not outweight the benefits of statin therapy | AACE-ACE, 2017 (22) | 1A |
|  | Routine non-invasive screening (angio-TC) is not recommended | NLA, 2011 (23)  IFHF, 2014 (9) | NA  3C |
|  | Adults with contraindications to statins: ezetimibe monotherapy is recommended | NICE, 2016 (26)  AACE-ACE, 2017 (22) | NA  2B |
|  | Adults with contraindications to statins: should be referred to a specialist | NLA, 2011 (23) | NA |
|  | Therapy: combination of lifestyle, statin treatment (with or without ezetimibe). | IFHF, 2014 (9)  NICE, 2016 (26)  AACE-ACE, 2017 (22) | 1A  NA  !A |
|  | when prescribing ezetimibe with a statin, ezetimibe should be prescribed on the basis of the lowest acquisition cost | NICE, 2016 (26) | NA |
|  | LDL apheresis should be considered in patients who cannot achieve LDL-cholesterol targets despite maximal drug therapy | NLA, 2011 (23)  IFHF, 2014 (9)  NICE, 2016 (26) | NA  2A  NA |
|  | Diet and drug therapy to lower LDL-cholesterol should be continued during LDL apheresis | IFHF, 2014 (9) | 2A |
|  | The efficacy, tolerability and safety of LDL apheresis must be regularly reviewed | IFHF, 2014 (9) | 3A |
|  | The effect of LDL apheresis on progression of atherosclerosis should be monitored according to clinical indications in FH patients with echocardiography, carotid ultrasonography and exercise stress testing | IFHF, 2014 (9) | 3B |
|  | Should recommend arterio-venous fistulae as the preferred method of access for people who are offered LDL apheresis | NICE, 2016 (26) | NA |
|  | Routine monitoring of the person iron’s status should be carried out and iron supplementation initiated if required in people who are receiving LDL apheresis | NICE, 2016 (26) | NA |
|  | ACE inhibitors should not be used in people treated with LDL apheresis | NICE, 2016 (26) | NA |
|  | People who are receiving LDL apheresis and blood-pressure lowering drug therapy should consider stopping it the morning of the LDL apheresis | NICE, 2016 (26) | NA |
|  | People undergoing LDL apheresis should stop warfarin therapy 4 days before and substitute it with EBWH. Anti-platelet therapy should continue | NICE, 2016 (26) | NA |
|  | Systematic cascade screening should be coordinated by a dedicated center and should not be carried out in primary care without central coordination | IFHF, 2014 (9) | 1A |
|  | Target: HDL  concentration > 40 mg/dl;  Target: TG  concentration < 150 mg/dl; | AACE-ACE, 2017 (22) | 1A |
| Normal CVR | Target: LDL-c < 100 mg/dl | ESC-EAS, 2016 (20)  IFHF, 2014 (9)  Descamps, 2011 (25)  ESC-EAS, 2016 (20)  TSLA, 2017 (27) | 1C  2C  IC  2C  1C |
|  | Therapy: aggressive statin therapy in combination with ezetimibe/resins if needed | ESC-EAS, 2016 (20)  SISA, 2014 (24) | 1C  NA |
|  | LDL apheresis should be considered when LDL  > 300 mg/dl | NLA, 2011 (23) | NA |
| High CVR | Target: LDL concentration  < 100 mg/dl | NLA, 2011 (23)  SISA, 2014 (24) | NA  NA |
|  | Therapy: aggressive statin therapy in combination with ezetimibe/resins if needed | NICE, 2016 (26) | NA |
|  | LDL apheresis should be considered when  LDL > 200 mg/dl | NLA, 2011 (23) | NA |
| Very high CVR | Target: LDL concentration  < 70 mg/dl | IFHF, 2014 (9)  SISA, 2014 (24)  TSLA, 2017 (27)  ESC-EAS, 2016 (20) | 2C  NA  1C  2C |
|  | Therapy: aggressive statin therapy in combination with ezetimibe/resins if needed | FHANCG, 2011 (25) SISA, 2014 (24) | A  NA |
|  | LDL apheresis should be considered when  LDL > 160 mg/dl | FHANCG, 2011 (25)  NLA, 2011 (23) | A  NA |
|  | Low dose aspirin (75-81 mg per day) should be considered | NLA, 2011 (23) | NA |
|  | Treatment with PCSK9 antibody should be considered | ESC-EAS, 2016 (20) | 2C |
| Without clinical CVD | Target: LDL concentration  < 70 mg/dl | AACE-ACE, 2017 (22) | 1A |
|  | Target: apoB concentration  < 80 mg/dl | AACE-ACE, 2017 (22) | 1A |
| With clinical CVD | Target: LDL concentration  < 70 mg/dl | SISA, 2014 (24)  IFHF, 2014 (9) | NA  2C |
|  | Target: LDL concentration  < 55 mg/dl | AACE-ACE, 2017 (22) | 1A |
|  | Target: apoB concentration  < 80 mg/dl | AACE-ACE, 2017 (22) | 1A |
|  | With symptoms of CHD immediately life-threatening should be referred to hospital as an emergency | NICE, 2016 (26) | NA |
|  | With symptoms of CHD not immediately life-threatening should be referred to a specialist | NICE, 2016 (26) | NA |
| with progressive ASCVD, or diabetes, or stage 3 or 4 CKD, or a history of premature ASCVD | Target: LDL c < 55 mg/dl | AACE-ACE, 2017 (22) | 1A |
|  | ADULT PATIENTS WITH POSITIVE (homozygous) TEST RESULT |  |  |
| All | Should be undertaken within a specialist centre | NICE, 2016 (26) | NA |
|  | liver transplantations should be considered as an option after drug therapy treatment and LDL apheresis | NICE, 2016 (26) | NA |
|  | Should be offered a referral for an evaluation of CHD | NICE, 2016 (26) | NA |
|  | Screening for coronary calcium score every 3 years | Al-Ashwal, 2015 (19) | NA |
|  | Screening for plaque formation every 5 years using low radiation computerised tomographic angiography | Al-Ashwal, 2015 (19) | NA |
|  | Carotid intima media thickness should ideally be assessed every 6 months | Al-Ashwal, 2015 (19) | NA |
|  | Stress testing is not recommended for assessment of atherosclerotic plaques | Al-Ashwal, 2015 (19) | NA |
|  | In clinical HoFH,  First-line treatment: diet/lifestyle + maximum tolerated  dose of high efficacy statins ± ezetimibe ± resin (If target is not achieved after 3 months with statins apheresis is recommended) | Al-Ashwal, 2015 (19) | NA |
|  | Smoking  should be avoided and exercise should be undertaken. | Al-Ashwal, 2015 (19) | NA |
|  | first-line treatment should be LA (preferred) or PEX | Al-Ashwal, 2015 (19) | NA |
|  | Apheresis should be started by  age of 5 years and no later than 8 years | Al-Ashwal, 2015 (19) | NA |
|  | If target is not achieved, new lipid-lowering therapies,  such as lomitapide could be added (to statins or apheresis) | Al-Ashwal, 2015 (19) | NA |
| With clinical CVD | Target: LDL c< 70 mg/dl | IFHF, 2014 (9)  SISA, 2014 (24)  Al-Ashwal, 2015 (19)  TSLA, 2017 | 1A  NA  NA  1C |
|  | Therapy: combination of lifestyle and drug therapy | IFHF, 2014 (9) | 1A |
|  | Lomitapide should be considered as adjunctive treatments to further lower LDLc | IFHF, 2014 (9) | 1C |
|  | Mipomersen should be considered as adjunctive treatments to further lower LDLc when Lomitapide is not tolerated | IFHF, 2014 (9) | 3C |
|  | LDL apheresis is indicated | IFHF, 2014 (9)  NICE, 2016 (26) | 1A  NA |
| Without clinical CVD | Target: LDL concentration < 100 mg/dl | TSLA, 2017 | 1C |
|  | Therapy: combination of lifestyle and drug therapy.  Lomitapide should be considered as adjunctive treatments to further lower plasma LDL-c levels | IFHF, 2014 (9) | 1C |
|  | Mipomersen should be considered as adjunctive treatments to further lower LDLc when Lomitapide is not tolerated | IFHF, 2014 (9) | 3C |
|  | LDL apheresis is indicated | FHANCG, 2011 (25)  NLA, 2011 (23)  SISA, 2014 (24)  IFHF, 2014 (9)  NICE, 2016 (26) | A  NA  NA  1A  NA |
|  | Assessment of 10-year risk is not recommended | NLA, 2011 (23) | NA |
|  | **Women with positive (heterozygous) test results** |  |  |
| With attempt to conceive | Should have pre-pregnancy counselling | FHANCG, 2011 (25)  NLA, 2011 (23)  IFHF, 2014 (9) | A  NA  2A |
|  | Should have appropriate advice on contraception | IFHF, 2014 (9)  NICE, 2016 (26) | 2A  NA |
|  | Should stop the lipid-modifying therapy 3 months before | FHANCG, 2011 (25)  IFHF, 2014 (9)  NICE, 2016 (26) | A  2A  NA |
|  | Should stop the lipid-modifying therapy 1 month before | NLA, 2011 (23) | NA |
| Pregnant | Who conceive while taking statins, should be advised to stop immediately and should be offered an urgent referral to a foetal assessment; they should also be given time to consider options | NLA, 2011 (23)  Al-Ashwal, 2015 (19)  NICE, 2016 (26) | NA  NA  NA |
|  | Should be offered assessment of coronary heart disease risk, particularly to exclude aortic stenosis | NICE, 2016 (26)  Al-Ashwal, 2015 (19) | NA  NA |
| During breast feeding | Statins should be discontinued | FHANCG, 2011 (25)  NLA, 2011 (23)  IFHF, 2014 (9)  Al-Ashwal, 2015 (19)  NICE, 2016 (26) | NA  NA  2A  NA  NA |
|  | Only resins should be considered as a drug treatment | NICE, 2016 (26) | NA |
|  | **Women with positive (homozygous) test results** |  |  |
| Pregnant | LDL apheresis should be considered | NLA, 2011 (23)  Al-Ashwal, 2015 (19) | NA  NA |
|  | Should be offered assessment of coronary heart disease risk, particularly to exclude aortic stenosis | NICE, 2016 (26) | NA |
|  | Who conceive while taking statins, should be advised to stop immediately and should be offered an urgent referral to a foetal assessment; they should also be given time to consider options | NICE, 2016 (26) | NA |
|  | **Children with positive (heterozygous) test result** |  |  |
| Without other CVRs | No diet before 2 years of age | Descamps, 2011 (25) | NA |
|  | Lifestyle, diet, no smoking, physical activity from 2 years of age | Descamps, 2011 (25) | NA |
|  | Statin therapy from 10 years of age | FHANCG, 2011 (25) | C |
|  | Target: LDL  concentration < 160 mg/dl | FHANCG, 2011 (25) | C |
|  | Target: LDL  concentration < 135 mg/dl | ESC-EAS, 2016 (20)  TSLA, 2017 | 2C  1C |
| With other CVRs | Statin therapy from 10 years of age  Target: LDL  concentration < 130 mg/dl  (reduction of LDL-C levels of 30% for Descamps, 2011 (25)) | NLA, 2011 (23)  FHANCG, 2011 (25)  Descamps, 2011 (25)  SISA, 2014 (24) | NA  A  NA  NA |
| All | Target: LDL  concentration < 100 mg/dl;  <130 mg/dl is borderline | AACE-ACE, 2017 (22) | D |
|  | Target: LDL  concentration < 100 mg/dl after 18 years | Descamps, 2011 (25) | NA |
|  | Treatment with statin after 18 years (with or without etezemibe of nicotinic acid) | Descamps, 2011 (25) | NA |
|  | Referral to a specialist and inform them that the treatment is lifelong | NICE, 2016 (26) | NA |
|  | Should be treated with statins considered at age 8 to 10 years | IFHF, 2014 (9)  ESC-EAS, 2016 (20) | 2B  2C |
|  | Should be treated with statins from 10 years of age | NICE, 2016 (26)  AACE-ACE, 2017 (22) | NA  4D |
|  | Should be treated with higher dose of statins or before 10 years of age in particular cases of family history of CHD in early adulthood | NICE, 2016 (26) | NA |
|  | Boys and girls should generally be treated at similar ages, although with a particularly adverse family of CHD and other major risk factors boys could be considered for earlier treatment with statins | IFHF, 2014 (9) | 2B |
|  | Primary preventive nutrition consisting of healthy lifestyle habits is recommended | AACE-ACE, 2017 (22) | 1A |
|  | To lower elevated plasma LDL-cholesterol generally requires a fat-modified, hearth healthy diet and statin, with the possible addition of ezetimibe or a bile acid sequestrants | IFHF, 2014 (9) | 1A |
|  | Referral to a specialist with expertise in FH | NICE, 2013  IFHF, 2014 (9) | NA  2A |
|  | Routine monitoring of growth and pubertal development should be considered | FHANCG, 2011 (25)  IFHF, 2014 (9)  NICE, 2016 (26) | B  1A  NA |
|  | Must receive advice on lifestyle modifications and non-lipid risk factors must be addressed.  Effective anti-smoking advice is mandatory | FHANCG, 2011 (25)  IFHF, 2014 (9)  SISA, 2014 (24) | A  2A  NA |
|  | Plasma levels of hepatic aminotransferases, creatine kinase and creatinine should be measured before starting pharmacotherapy. All patients receiving statins should have hepatic aminotransferases monitored; creatine kinase should be measured when musculoskeletal symptoms are reported; creatinine should be monitored in those with kidney disease | FHANCG, 2011 (25)  IFHF, 2014 (9) | A  2A |
|  | Use statins licensed for clinical use in this age group | IFHF, 2014 (9)  NICE, 2016 (26) | 1C  NA |
|  | Evaluation of CHD to detect clinically significant disease and referral should not be routinely offered | NICE, 2016 (26) | NA |
| Who are intolerant of statins | Should be offered other lipid-modifying drug therapies capable of reducing LDL concentration (such as bile acid sequestrants [resins], fibrates or ezetimibe) | IFHF, 2014 (9) | 1A |
| Girls | Should have pre-pregnancy counselling with appropriate advice on contraception | IFHF, 2014 (9) | 3A |
|  | **Children with positive (homozygous) test results** |  |  |
| All | Should always be managed by a lipid specialist | NLA, 2011 (23)  IFHF, 2014 (9) | NA  2A |
|  | Should be offered a referral for an evaluation of CHD | NICE, 2016 (26) | NA |
|  | They need special attention from the first year of life | NLA, 2011 (23) | NA |
|  | Drug therapy should be considered before LDL apheresis | NICE, 2016 (26) | NA |
|  | Who are intolerant to statins, should be offered other treatments with bile acide sequestrants, fibrates or ezetimibe | NICE, 2016 (26) | NA |
|  | LDL concentration target:  < 135 mg/dL | NLA, 2011 (23)  Al-Ashwal, 2015 (19)  TSLA, 2017 | NA  NA  1C |
|  | Therapy: combination of lifestyle, statin treatment (with or without ezetimibe) | FHANCG, 2011 (25)  NLA, 2014 | A  NA |
|  | LDL apheresis is indicated | FHANCG, 2011 (25)  NLA, 2011 (23)  SISA, 2014 (24)  IFHF, 2014 (9) | A  NA  NA  2A |
|  | LDL apheresis should be considered by the age of 5 and no later than 8 years | IFHF, 2014 (9) | 2A |
|  | Diet and drug therapy to lower LDL-cholesterol should be continued during LDL apheresis | IFHF, 2014 (9) | 2A |
|  | The efficacy, tolerability and safety of LDL apheresis must be regularly reviewed | IFHF, 2014 (9) | 3A |
|  | Lomitapide and mipomersen should be considered as adjunctive treatments to further lower plasma LDL concentration levels | NLA, 2011 (23)  IFHF, 2014 (9) | NA  3C |
|  | Liver transplantation should be considered for younger patients who have rapid progression of atherosclerotic or aortic stenosis, cannot tolerate LDL apheresis or when LDL cholesterol level can’t be adequately lowered | IFHF, 2014 (9) | 3B |
|  | | | |

**Definitions of cardiovascular risk (CVR)**

FHANCG, 2011 (21):

low CVR: absence of other major risk factors (smoke, obesity, hypertension, diabetes)

intermediate CVR: one risk factor or subclinic evidences of CVD

high CVR: patients with CVD

NLA, 2011 (23):

moderate high CVR (> 2 Risk Factors & 10-20% CHD risk)

high CVR: CHD risk equivalent

very high CVR: clinically evident CHD or other atherosclerotic CVD, diabetes, a family history of very early CHD (in men , 45 years of age and women , 55 years of age), current smoking, two or more CHD risk factors, or high levels of Lp(a)

IFHF, 2014 (9):

normal CVR: absence of CVD end of other major risk factors (unspecified)

very high CVR: patients with CVD or diabetes

SISA, 2014 (24):

very high CVR: patients with CVD or with high levels of Lp

ESC-EAS, 2016 (20):

normal CVR: no other risk factors

very high CVR: patients with CVD, with other risk factors (unspecified), with a family history of CVD, with intolerance to statins, or with high levels of Lp(a)

TSLA, 2017 (27):

normal CVR: absence of CVD end of other major risk factors (unspecified)

very high CVR: patients with CVD or diabetes.
